# A polymicrobial biofilm model for testing the antimicrobial potential of a nisin-biogel for canine periodontal disease control

**DOI:** 10.1186/s12917-020-02646-3

**Published:** 2020-12-02

**Authors:** Eva Cunha, Sandra Rebelo, Carla Carneiro, Luís Tavares, Luís Miguel Carreira, Manuela Oliveira

**Affiliations:** CIISA-Centro de Investigação Interdisciplinar em Sanidade Animal, Faculdade de Medicina Veterinária, Universidade de Lisboa, Avenida da Universidade Técnica, 1300-477 Lisboa, Portugal

**Keywords:** Periodontal disease, Polymicrobial biofilm, Fluorescent *In situ* Hybridization, Nisin-biogel

## Abstract

**Background:**

Periodontal disease (PD) in dogs is prompted by the establishment of a polymicrobial biofilm at the tooth surface and a subsequent host inflammatory response. Several strategies may be used for PD control, including dental hygiene home care procedures, like toothbrushing, special diet and chew toys that reduce dental plaque accumulation, or professional periodontal treatments. Aiming at PD control, a biogel composed by nisin and guar-gum was previously developed. This work aimed to establish an *in vitro* model mimicking the PD-associated biofilms and to evaluate the nisin-biogel inhibitory activity against this polymicrobial biofilm by determining its Minimum Biofilm Inhibitory (MBIC) and Eradication Concentrations (MBEC). Bacterial species tested included *Neisseria zoodegmatis* CCUG 52598T, *Corynebacterium canis* CCUG 58627T, *Porphyromonas cangingivalis* DSMZ VPB 4874, *Peptostreptococcus canis* CCUG 57081 and an *Enterococcus faecalis* isolate belonging to a collection of oral bacteria obtained from dogs with PD. Before establishing the biofilm, coaggregation between species was determined by optical density measurement after 2 and 24 hours. Nisin-biogel MBIC and MBEC values regarding the polymicrobial biofilm were determined using a modified version of the Calgary biofilm pin lid device, after confirming the presence of the five bacterial species by Fluorescent *In Situ* Hybridization.

**Results:**

Only 40% of the bacterial dual suspensions were able to coaggregate at 2 hours, but all species tested exhibited a coaggregation percentage higher than 30% at 24 hours. It was possible to establish a 48 h polymicrobial biofilm model composed by the five bacterial species selected. This model was used to determine nisin-biogel MBIC (26.39 ± 5.89 µg/mL) and MBEC (62.5 ± 27.73 µg/mL) values.

**Conclusions:**

Our results showed that the nisin-biogel can inhibit and eradicate PD multispecies biofilms. As this *in vitro* model mimics an *in vivo* periodontal polymicrobial biofilm, our results reinforce the potential of the application of nisin-biogel for canine PD control.

## Background

Periodontal disease (PD) is one of the most common inflammatory disease in dogs, with reports suggesting that 80 to 85% of dogs with more than 2 years will develop this disease [1–3]. PD is initiated by the formation of a polymicrobial biofilm in the teeth surface (dental plaque), which induces a local inflammatory response, leading to gingivitis and/or several degrees of periodontitis [3].

The formation of dental plaque is a multi-stage process, that begins by the formation of a pellicle in the teeth surface. This pellicle is mainly composed by salivary glycoproteins, that cover the dental surface and allow oral bacteria to adhere and establish the biofilm [3, 4].

Several bacteria are reported to be present at different stages of dental plaque formation. Early or primary colonizers are responsible for the initial formation of the biofilm and include aerobic bacteria, such as *Bergeyella*, *Neisseria*, *Moraxella*, *Corynebacterium* and *Stenotrophomonas* species, that interact with the pre-formed pellicle, with other bacteria from the same species (auto-aggregation) and with bacteria from different species (coaggregation) [4–8]. They also participate in the formation of the biofilm matrix, composed by salivary glycoproteins, extracellular polysaccharides, lipids and cellular debris, that hold the biofilm and facilitate the adherence of additional bacteria [3, 4, 9].

Contrary to the human dental plaque, mostly composed by Gram-positive strains, the canine dental plaque microbiome follows an opposite trend, evolving from a majority of Gram-negative aerobic species to Gram-positive anaerobic species [6, 7, 10]. In addition to the primary colonizers, one of the most abundant bacterial species in the oral canine microbiome and with an important role in PD development is *Phorphyromonas* spp. [6, 7, 10–14]. These bacteria are present in the healthy oral cavity but also in animals with gingivitis or perio-dontitis. [6, 7]. Its ability to survive in the oral cavity, regardless of the PD stage, and capacity to produce several virulence factors renders *Porphyromonas* sp. one of the keystone pathogens in PD research [4, 8, 14].

After initial dental plaque formation, other anaerobic bacteria adhere to the formed biofilm, replacing some of the early colonizers and contributing to PD progression. As a late colonizer, *Peptostreptococcus canis*, an anae-robic. Gram-positive strain, is being strongly related to high grade periodontitis in dogs [8, 9, 13, 15]. Other important bacteria frequently related to periodontitis in dogs are *Treponema denticola*, *Prevotella intermedia*, *Tannerella forsythia*, *Actinomices canis*, *Bacteroides* sp., *Campylobacter rectus*, *Fusobacterium* sp., *Pseudomonas* sp., *Staphylococcus* sp., *Streptococcus* sp. and *Capnocytophaga* sp. [3, 4, 9, 11, 12, 16, 17]. Moreover, some oral bacteria that participate in PD development, such as *Enterococcus faecalis*, can reach the bloodstream and be associated with PD-related systemic consequences [18].

Considering the high prevalence of canine PD, effective control measures are essential for its prevention and treatment. Bacterial dental plaque control is crucial. Removal of dental plaque and inhibition of its formation can be achieved by a combination of dental hygiene homecare procedures and application of several oral products, use of specific diet and chew toys, and regular professional periodontal procedures [19–21]. Toothbrushing is the most effective method for daily plaque control by mechanically disruption of the dental plaque, being considered the gold standard method for PD control [19, 22]. As a complementary measure of toothbrushing, chemical plaque control agents are also useful. These compounds disrupt the polymicrobial biofilm, or prevent its formation [23]. Chlorhexidine is the most frequent antiplaque agent used, being applied as a solution to irrigate the oral cavity before dental scaling or surgical procedures, showing good activity against oral pathogens [24]. Recently, it was been proposed that nisin-biogel is a promising compound to inhibit and eradicate enterococcal PD biofilms in dogs [1]. This compound combines the antimicrobial activity of nisin, and the delivery capacity of the plant-derived polysaccharide guar gum (biogel) [1]. Nisin, produced mainly by *Lactococcus lactis*, is an antimicrobial peptide with antimicrobial activity against Gram-positive bacteria, some Gram-negative, and multidrug resistant bacteria, both in their planktonic and biofilm forms [1, 25, 26]. Its antibacterial ability results from the interaction with the lipid II in the bacterial cytoplasmic membrane, leading to membrane pore formation and inhibition of cell wall synthesis [1]. Besides the demonstrated antimicrobial activity of the nisin-biogel against oral pathogens [1, 26], this compound also keeps its activity in the presence of saliva, present no cytotoxicity up to 200 µg/mL, and it has rarely been involved in resistance development [26, 27]. To confirm its ability to control the dental plaque, the main goal of this work was to develop an *in vitro* polymicrobial biofilm, composed by strains associated to the different stages of PD evolution, in order to evaluate nisin-biogel inhibitory activity against the polymicrobial biofilm, by determining the Minimum Biofilm Inhibitory (MBIC) and Eradication Concentrations (MBEC). The polymicrobial biofilm model was established using five strains, namely *Neisseria zoodegmatis* CCUG 52598T, *Corynebacterium canis* CCUG 58627T, *Enterococcus faecalis* clinical isolate, *Porphyromonas cangingivalis* DSMZ VPB 4874 and *Peptostreptococcus canis* CCUG 57081.

## Results

### Bacterial coaggregation

Coaggregation ability between isolates from the collection of enterococci from dogs with PD and the early colonizers *N. zoodegmatis* CCUG 52598T and *C. canis* CCUG 58627T was evaluated at 2 and 24 hours. Results are presented in Table 1. None of the enterococci presented more than 30% of aggregation ability with both *N. zoodegmatis* CCUG 52598T and *C. canis* CCUG 58627T, in both incubation periods. The *Enterococcus* isolates that presented a coaggregation with *N. zoodegmatis* CCUG 52598T and *C. canis* CCUG 58627T higher than 30%, at 24 hours of incubation were: M3b, M4c, M15b, M15d, M21c, M25a, M25c, M29b and M32a. Considering the increasing aggregation ability over time and the aggregation value, the *E. faecalis* M32a isolate was selected to be used in the following experiments.

**Table 1:** Coaggregation ability between the enterococci collection, *Neisseria zoodegmatis* CCUG 52598T and *Corynebacterium canis* CCUG 58627T

Isolate ID	*Neisseria zoodegmatis* CCUG 52598T		*Corynebacterium canis* CCUG 58627T	
	**Coaggregation ability (%)**			
	**2 h**	**24 h**	**2 h**	**24 h**
M2b	30.65 ± 2.60	55.96 ± 0.07	7.94 ± 4.52	1.08 ± 1.07
M2c	7.04 ± 2.30	34.61 ± 3.24	-0.79 ± 14.77	16.49 ± 5.21
M3b	4.30 ± 0.85	31.17 ± 6.36	6.33 ± 1.12	37.87 ± 37.76
M3d	-0.10 ± 2.13	16.81 ± 0.09	13.57 ± 7.02	18.26 ± 2.2
M4a	5.85 ± 0.63	14.67 ± 6.16	23.98 ± 5.86	20.19 ± 5.76
M4c	44.78 ± 0.57	35.99 ± 0.50	26.78 ± 8.20	36.14 ± 9.55
M15b	12.32 ± 0.77	39.48 ± 2.94	23.48 ± 21.03	60.89 ± 20.89
M15d	12.05 ± 2.86	40.17 ± 0.34	27.04 ± 1.59	39.40 ± 14.12
M21a	7.43 ± 0.17	13.72 ± 1.00	12.05 ± 13.56	18.58 ± 8.20
M21c	1.77 ± 4.48	36.6 ± 1.67	-14.26 ± 18.26	40.33 ± 19.19
M23a	8.48 ± 1.08	34.79 ± 0.48	20.28 ± 1.92	24.32 ± 1.80
M23c	5.89 ± 0.47	40.61 ± 3.36	24.57 ± 8.74	22.31 ± 11.48
M25a	9.91 ± 3.72	42.33 ± 3.93	9.56 ± 1.68	38.20 ± 16.02
M25c	4.18 ± 0.83	36.61 ± 6.15	-3.66 ± 7.71	33.04 ± 21.65
M29b	44.76 ± 0.07	40.53 ± 1.19	3.96 ± 17.77	38.85 ± 8.47
M32a	19.62 ± 0.41	44.84 ± 4.39	35.57 ± 7.24	60.02 ± 10.57
M32b	-3.82 ± 6.99	21.81 ± 0.41	3,02 ± 3,00	7.44 ± 7.21

Legend: Determination of the coaggregation ability (%) between the isolates from the enterococci collection obtained from the oral cavity of dogs with PD, and *Neisseria zoodegmatis* CCUG 52598T and *Corynebacterium canis *CCUG 58627T, after 2 and 24 hours. ID – identification. % - percentage (mean ± standard deviation). h – hours

Then, evaluation of auto-aggregation and coaggregation abilities were performed between all five bacteria selected for the biofilm model: *N. zoodegmatis* CCUG 52598T, *C. canis* CCUG 58627T, *E. faecalis* (M32a), *P. cangingivalis* DSMZ VPB 4874 and *P. canis* CCUG 57081. Results are presented in Table 2. Only 40% (4/10) of the bacterial dual suspensions showed coaggregation higher than 30% at 2 hours. All bacterial dual suspensions exhibited a percentage of coaggregation higher than 30% at 24 hours.

**Table 2:** Auto-aggregation and coaggregation ability (%) determination

Bacterial species	Coaggregation (%)	
	**2 h**	**24 h**
*N. zoodegmatis*	7.07 ± 1.30	33.27 ± 1.45
*N. zoodegmatis + E. faecalis*	19.62 ± 0.41	44.84 ± 4.39
*N. zoodegmatis + C. canis*	48.99 ± 15.12	59.12 ± 6.01
*N. zoodegmatis + P. canis*	18.06 ± 1.11	48.79 ± 0.57
*N. zoodegmatis + P. cangingivalis*	16.57 ± 6.80	46.47 ± 8.77
*E. faecalis*	6.82 ± 4.8	28.94 ± 6.42
*E. faecalis + C. canis*	35.57 ± 7.24	60.02 ± 10.57
*E. faecalis + P. canis*	9.00 ± 6.41	42.33 ± 1.73
*E. faecalis + P. cangingivalis*	16.77 ± 6.28	46.24 ± 17.03
*C. canis*	83.74 ± 2.8	82.42 ± 5.16
*C. canis + P. canis*	43.14 ± 11.91	62.77 ± 9.23
*C. canis + P. cangingivalis*	46.58 ± 18.60	60.24 ± 16.55
*P. canis*	24.48 ± 16.69	49.37 ± 6.58
*P. canis + P. cangingivalis*	25.27 ± 12.75	59.25 ± 20.25
*P. cangingivalis*	14.31 ± 5.83	45.09 ± 1.45

Legend: Auto-aggregation and coaggregation ability (%), after 2 and 24 hours, between *Neisseria zoodegmatis* CCUG 52598T, *Corynebacterium canis *CCUG 58627T,* Enterococcus faecalis* (M32a), *Porphyromonas cangingivalis* DSMZ VPB 4874 and *Peptostreptococcus canis* CCUG 57081. % - percentage (mean ± standard deviation). h – hours

### Polymicrobial biofilm formation and Fluorescence in situ hybridization (FISH)

**Fig. 1:**
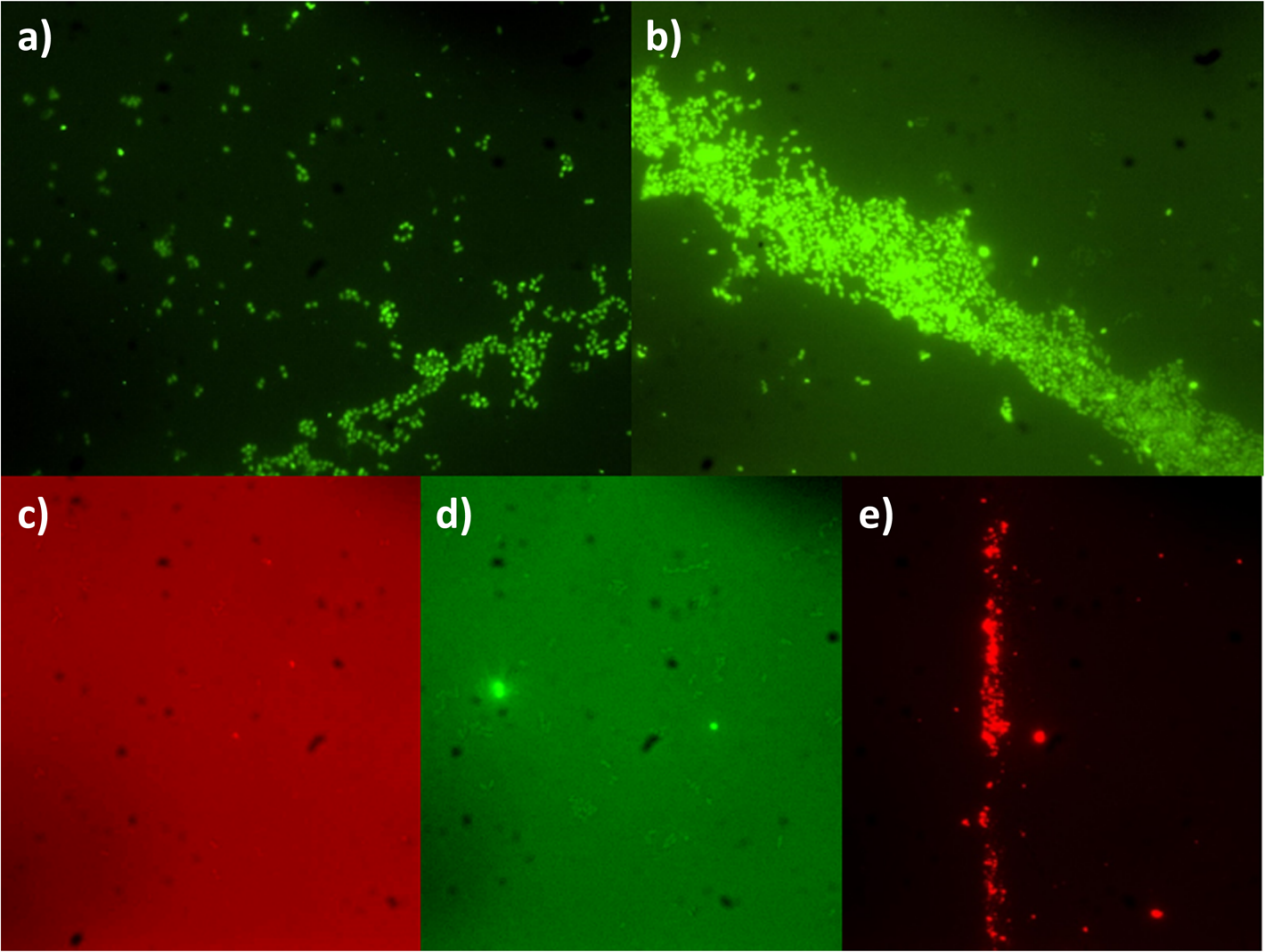
Bacterial detection through the different specific FISH protocols applied to samples of the polymicrobial biofilm. Legend: **a)** *Enterococcus faecalis *(M32a); **b)** *Porphyromonas cangingivalis.* DSMZ VPB 4874; **c)** *Neisseria zoodegmatis* CCUG 52598T; **d)*** Corynebacterium canis* CCUG 58627T; and **e)** *Peptostreptococcus canis* CCUG 57081

The protocol used allowed to form polymicrobial biofilms, as all five bacterial species were detected by FISH in the 48 h polymicrobial biofilm (Fig. 1). Species representativity in the biofilm model varied, being *N. zoodegmatis* CCUG 52598T the one present in the lower concentration, followed by *C. canis* CCUG 58627T and *P. canis* CCUG 57081. *E. faecalis* (M32a) and *P. cangingivalis* DSMZ VPB 4874 were the most abundant species in the biofilm model.

### Determination of the nisin-biogel minimum biofilm inhibitory concentration (MBIC) and minimum biofilm eradication concentration (MBEC) regarding the polymicrobial biofilm

The polymicrobial biofilm was susceptible to nisin-biogel and chlorhexidine solutions. Considering the control chlorhexidine, the mean MBIC value was 0.00094%, and the mean MBEC value was 0.00321% ± 0.002. The mean MBIC value of nisin-biogel against the polymicrobial biofilm was 26.39 ± 5.89 µg/mL, and the mean MBEC value determined was 62.5 ± 27.73 µg/mL.

## Discussion

Oral health is undoubtedly a relevant topic in veterinary medicine. With a prevalence that can be higher than 80%, PD represents one of the most widespread inflammatory disease in dogs [2, 3]. The study of the canine oral microbiome, as well as new drugs, tools and metho-dologies to prevent, treat and mimic PD, are of major relevance [1].

The main goal of this work was to develop an *in vitro* model of a canine dental plaque biofilm to test the antimicrobial ability of a new compound, the nisin-biogel, in conditions that better simulate what is observed *in vivo*.

First, it was necessary to select a group of bacteria with relevant roles in PD development and progression, and to understand if they were able to co-aggregate. Two early colonizers were chosen, *N. zoodegmatis* CCUG 52598T and *C. canis* CCUG 58627T, and used to select one isolate from our enterococci collection, obtained from the oral cavity of dogs with PD [7, 28]. From the 17 enterococci tested, nine presented a coaggregation ability higher than 30% at 24 hours with both primary colonizers (Table 1). Combining an increasing coaggregation ability between 2 and 24 hours and the better coaggregation result at 24 hours, *E. faecalis* M32a was selected to be included in the biofilm model experiment. Enterococci are usually present in the canine oral cavity, having the capacity of becoming opportunistic pathogens. Being facultative anaerobes, enterococci can persist in a mature biofilm and may facilitate the adhe-rence of other bacteria to the biofilm, also having a possible link to the development of PD-related systemic diseases [18, 28].

After that, auto-aggregation and coaggregation ability of the selected enterococci was tested using two other bacterial strains, *P. cangingivalis* DSMZ VPB 4874 and *P. canis* CCUG 57081. *P. cangingivalis* is a highly abundant bacteria in the oral cavity of healthy dogs and in animals with inflamed periodontal tissues [6, 10, 13, 14], since its metabolic flexibility supports its survival in both environments [4, 13]. On another hand, *P. canis* is a late colonizer frequently identified in severe PD cases [9, 15].

The five species tested showed a coaggregation ca-pacity higher than 30% after 24 hours. Usually, coaggregation interactions are highly specific and involve recognition of receptors between bacteria, with involvement of adhesins and protein-saccharide interactions due to the presence of flagella and pili [8, 29, 30]. Coaggregation can also actively modulate gene expression, being one important factor on biofilm formation [29]. In our study, it was possible to observe that, after only 2 hours of incubation, the two early colonizers (*N. zoodegmatis* CCUG 52598T and *C. canis* CCUG 58627T) showed the higher coaggregation capacity, followed by the dual suspension of *C canis* CCUG 58627T and *P. cangingivalis* DSMZ VPB 4874. Then, after 24 hours of incubation, *C. canis* CCUG 58627T also presented a high coaggregation ability, with *P. canis* CCUG 57081, *P. cangingivalis* DSMZ VPB 4874 and *N. zoodegmatis* CCUG 52598T. Similarly to our study, Elliot et al. (2006) [5] and Holcombe at al. (2014) [7] also showed that *Corynebacterium* sp. can co-aggregate with several oral bacteria, being an important pioneer in oral biofilms. As described, *P. cangingivalis* has an important role in PD development [4, 8], resulting from its high coaggregation ability with both early and late colonizers, at 2 and 24 hours, as showed in this work. In fact, coaggregation evaluation is a very important step to predict the behaviour of bacteria in a biofilm community.

Considering dental plaque establishment, and to simulate the salivary pellicle present at the teeth surface, the pegs used to form the polymicrobial biofilms, were incubated 2 hours in CAS. This canine artificial saliva was prepared as described by Sanguersrmi et al. (2017) [8], with several components that aim to simulate the biochemical composition of canine saliva. Then, the *in vitro* oral biofilm model was established using a combination of five strains, as previously described. Considering the low oxygen tension in the subgingival pocket, biofilms were established using a microaerophilic atmosphere to simulate the dental plaque environment and facilitate the adherence of all strains, which present distinct res-piratory requirements [31, 32].

Polymicrobial biofilm evaluation was performed by Fluorescence *In Situ* Hybridization (FISH). This technique is an easy to perform and quick method, allowing the identification of microbial populations in a complex community setting [33, 34]. FISH is based on specific hybridization of labelled oligonucleotide probes, with complementary rRNA target sequences present within a permeabilized and morphologically intact bacterial cell [33]. Specific probes for each of the five species present in the biofilm model were selected targeting the bacterial 16S rRNA [33]. All five species were identified at the biofilm model after FISH evaluation. However, some diffe-rences in bacterial representativity were observed in this study. In fact, some reports describe that early colonizers are essential in the initial biofilm establishment, but with the PD progression, they suffer a reduction, being replaced by secondary or late colonizers, which is in accordance with our results [11, 13]. This *in vitro* polymicrobial biofilm is a very interesting model for testing new drugs, and the methodology applied in this study can also be used to develop distinct polymicrobial models.

Considering the high prevalence of PD in dogs, control measures are essential to reduce its impact on animal health. PD prevention can be achieved by home oral hygiene procedures and regular professional periodontal evaluation, with the establishment of therapeutic protocols focusing on the prevention and removal of dental plaque [19, 20, 35]. PD treatment includes non-surgical techniques, aiming at the removal of factors that promote disease progression, and surgical procedures that promote periodontium regeneration [26]. In both cases antimicrobial therapy may be necessary depending on case severity [19, 21, 35]. In order to reduce antimicrobial use, a nisin-biogel was recently proposed as a pro-mising compound to be used in canine PD control [1, 27]. The authors showed that the nisin-biogel can inhibit and eradicate canine enterococcal-dental plaque biofilms [1]. In this work, the nisin-biogel was able to inhibit and eradicate oral polymicrobial biofilms at concentrations two-fold higher than the MBIC values and 3 folds higher than the MBEC values, previously determined for mono-species biofilms [1]. Likewise, other reports also describe nisin as having an *in vitro* antimicrobial activity against several dental plaque bacteria, reinforcing its potential for PD control [25, 26]. The potential cytotoxicity of nisin and nisin-biogel has already evaluated regarding several eukaryotic cells, revealing no toxicity up to 200 µg/mL [26, 27], and the European Food Safety Authority have defined an ADI of 1 mg/Kg/day for nisin use as a food additive [36]. All these points support the safety of nisin in a potential dental topical application *in vivo*.

In addition, our study showed that the polymicrobial biofilm was inhibited and eradicated by chlorhexidine, at concentrations lower than 0.12%, which is the currently concentration recommended to be used in Veterinary odontology [24]. Chlorhexidine is an antiseptic used in solution to irrigate the oral cavity before dental scaling or surgical procedures, showing good activity against oral pathogens [24]. Some products containing chlorhexidine are available to use in PD control [37]. However, it is described that chlorhexidine presents negative effects when used as a prolonged therapy, such as taste loss, pigmentation of the enamel or lesions of the oral mucosa [37]. In addition, oral bacteria may be resistant to chlorhexidine or present a cross resistance profile to chlorhexidine and several antimicrobials [38]. chlorhexidine should not be applied for long periods, not being recommended for PD prevention [37]. Considering that it is of major importance to develop new products, such as the nisin-biogel, to be used as a regular approach for PD control, aiming at reducing antimicrobial therapy.

In conclusion, the *in vitro* model of a periodontal polymicrobial biofilm developed, aiming at mimicking the *in vivo* conditions present in dogs oral cavity, allowed to observe that the nisin-biogel developed by our research team can be effective against multi-species biofilms, reinforcing its potential for controlling a relevant disease of these animals.

## Conclusions

Periodontal disease is one of the most common inflammatory disease in dogs, being caused by a polymicrobial biofilm formed in the teeth surface. Early colonizers, such as *N. zoodegmatis* and *C. canis*, adhere to the sali-vary pellicle in the teeth surface and allow the aggregation of secondary and late colonizers, to form a mature biofilm. In this work it was possible to develop an *in vitro* model of a periodontal polymicrobial biofilm, composed by five bacterial strains frequently present in dog’s dental plaque. In addition, this model was used to evaluate the inhibitory activity of a nisin-biogel, developed by our research team, allowing to observe that the biogel can inhibit and eradicate multi-species biofilms, reinforcing its potential to be used in the control of dogs PD.

## Methods

### Bacterial collection and culture conditions

Bacterial reference strains *Neisseria zoodegmatis* CCUG 52598T (from a human wound caused by a dog bite), *Corynebacterium canis* CCUG 58627T (from a human wound caused by a dog bite), *Peptostreptococcus canis* CCUG 57081 (from a canine dental plaque) and *Porphyromonas cangingivalis* DSMZ VPB 4874 (from a canine periodontal pocket) were used in this study. Each strain was selected due to their association to different stages of PD progression.

A collection of 17 biofilm-producer enterococci, obtained from the oral cavity of dogs diagnosed with PD was also used to select one isolate to be included in the formation of the five-species polymicrobial biofilm model [28].

*N. zoodegmatis* CCUG 52598T, *C. canis* CCUG 58627T and the enterococci were routinely grown on Brain Heart Infusion (BHI) agar plates (VWR, Belgium) under aerobic conditions for 24 hours at 37ºC. *P. canis* CCUG 57081 was grown on Chocolate Agar plates (VWR, Belgium) under anaerobic conditions for 48 hours at 37ºC. *P. cangingivalis* DSMZ VPB 4874 was grown on Columbia Blood agar plates (VWR, Belgium) under anaerobic conditions for 48 hours at 37ºC. All bacteria were also grown on *Brucella* Broth medium (Liofilchem, Italy), supplemented with hemin (5 µg/mL) (Sigma-Aldrich, USA) and vitamin K_1_ (1 µg/mL) (Liofilchem, Italy) [39].

### Nisin- biogel solutions

A nisin stock solution (1000 µg/mL, 40 000 IU/mL) (2.5% purity Sigma-Aldrich, USA) and a 1.5% guar-gum biogel (w/v) (Sigma-Aldrich, USA) solution were prepared as described elsewhere [1].

Nisin stock solution was diluted in sterile distilled water to achieve the following concentrations: 750, 625, 500, 375, 250, 125, 50, 25 and 12.5 µg/mL. Then these solutions were incorporated within the guar-gum gel in a proportion of 1:1, to obtain a 0.75% gel (w/v). Working solutions were kept at 4ºC during the study.

### Chlorhexidine solutions

A stock solution of chlorhexidine gluconate at 4% (w/v) (AGA, Portugal) was diluted in sterile distilled water and used as a control, as it is described as the drug of choice for PD control [24, 37]. Two-fold dilutions from 0.24–0.00047% were tested, according to the concentrations used in canine odontology [24, 37].

### Canine artificial saliva

Preparation of canine artificial saliva (CAS) was performed as described by Sanguansermsri et al. (2018) [8]. CAS was composed (per liter) by 1 g Lab Lemco Powder (Thermo Fisher Scientific, Denmark), 2 g yeast extract (Sigma-Aldrich, USA), 5 g proteose peptone (Merk, Germany), 2.5 g hog gastric mucin (Sigma-Aldrich, USA), 2.34 g NaCl, 1.5 g KCl, 0.1 g CaCl_2_ and 1.25 mL of 40% urea. The solution was sterilized by autoclave, except for urea that was filtered using a 0.22 µm cellulose acetate membrane filter and then added to the remaining components.

### Bacterial coaggregation

Coaggregation between bacterial strains was evaluated as described by Datta et al. (2017) [30] and Sanguansermsri et al. (2018) [8], with some modifications. Aerobic bacteria (*N. zoodegmatis* CCUG 52598T, *C. canis* and enterococci CCUG 58627T) were grown in 5 mL BHI broth at 37ºC for 24 hours, and anaerobic bacteria (*P. cangingivalis* DSMZ VPB 4874 and *P. canis* CCUG 57081) in 5 mL of supplemented *Brucella* Broth under anaerobic conditions for 48 hours at 37ºC. Then cells were harvested by centrifugation at 5000 g for 7 minutes at 4 °C and suspended in coaggregation buffer (1 mmol L^− 1^ Tris–HCl pH 8, 150 mmol L^− 1^ NaCl, 0.1 mmol L^− 1^ CaCl_2_.2H_2_O, 0.1 mmol L^− 1^ MgCl_2_ and 0.02% NaN_3_). The optical density at 600 nm (OD600) of each suspension was adjusted to 1.

Equal volumes of each bacterial suspension (1 mL) were mixed and vortexed for 30 seconds, after which the OD600 was measured (OD1). Then, the dual suspension was centrifuged for 2 minutes at 650 g and incubated at room temperature for 2 hours, afterwhich the OD600 of 0.2 mL of the upper layer was measured (OD2). This procedure was repeated after 24 hours (OD24). The percentage coaggregation was assessed using the following formula:

$$ \%\mathrm{Coaggregation}=\frac{\mathrm{OD}1-\mathrm{OD}2\left(\mathrm{or}\;\mathrm{OD}24\right)}{\mathrm{OD}1}\mathrm{x}\ 100 $$

A percentage higher than 30% was considered as a coaggregation indicator [30].

Coaggregation between enterococci isolates (n = 17) and *N. zoodegmatis* CCUG 52598T and *C. canis* CCUG 58627T, was determined to select the enterococci with the higher coaggregation ability to be used in the further protocols. Afterwards, coaggregation of the selected isolates with the anaerobic strains was determined, as well as auto-aggregation between the five bacterial species.

The experiments were repeated three times in independent days.

### Polymicrobial biofilm formation

A polymicrobial biofilm composed by five bacterial species, including *N. zoodegmatis* CCUG 52598T, *C. canis* CCUG 58627T, *E. faecalis* clinical isolate, *P. cangingivalis* DSMZ VPB 4874 and *P. canis* CCUG 57081, was performed, based on the protocol described by Vandeplassche et al. (2017) [32] and Sanguansermsri et al. (2018) [8] with some modifications. To achieve that, a modified version of the Calgary Biofilm Pin Lid Device and a microaerophilic environment (Merk, Germany) were used. Briefly, 40 µL of a 10^7^ CFU/mL bacterial suspension, from each strain, in supplemented *Brucella* broth medium were deposited in the wells of a 96-well microplate (Nunc™, Thermo Scientific). Then, a peg lid (Nunc™ Immuno TSP Lids, Thermo Scientific™), previously incubated for 2 hours in CAS, was applied in the microplate. After a 48 h incubation at 37 °C, in microaerophilic conditions, pegs were washed three times in 0.9% NaCl, transferred to a new microplate filled with fresh *Brucella* broth, sealed and incubated in an ultrasonic bath (Gramt, Ultrasonic Bath, MXB14) for 15 min at high frequency (50–60 Hz) [1]. Afterwards, the peg lid was replaced by a conventional one, and the microplate was incubated at 37 °C, for 48 hours, in microaerophilic conditions. Finally, bacterial suspensions from each well were evaluated by Fluorescence *In Situ* Hybridization (FISH) to confirm the presence of the five bacterial strains in the polymicrobial biofilm.

### Fluorescence In Situ Hybridization (FISH)

The FISH protocol was performed as described by Oliveira et al. (2006) [40] with some modifications. Teflon slides (Heinz Herenz, Hamburg, Germany) were used as hybridization supports. Before hybridization, slides were washed in ethanol, incubated in a 2% 3-trimethoxysilylpropilamine solution (Merck, Germany) in acetone (PanReac AppliChem, USA) for 1 min, twice in acetone for 1 min and washed in distilled water [40].

Then, 10 µl of the bacterial suspensions originated from the polymicrobial biofilms were placed in the wells of the slide. After air-drying, suspensions were fixed with a 4% paraformaldehyde (w/v) solution in PBS for 4 hours at room temperature. After fixation, suspensions were dehydrated with ethanol at 50, 80 and 96%, during 3 min at each concentration, permeabilized with lysozyme (0.5 µg/ mL) (Sigma-Aldrich, USA) during 20 min at room temperature, and dehydrated again. Afterwards, 10 µl of hybridization buffer (0.9 M NaCl, 20 mMTris– HCl, pH 7.2, 0.01% SDS) were added, containing 5 ng/ml of each specific probe (STABVIDA, Portugal) mentioned in Table 3. Slides were incubated in a humid chamber (Omnislide Thermal Cycling Block, Hybaid Omnislide System, Thermoelectron Corporation, USA) during 3 h, at 46ºC for *N. zoodegmatis* CCUG 52598T, *E. faecalis* and *P. cangingivalis* DSMZ VPB 4874 detection, at 48ºC for *C. canis* CCUG58627T detection, and at 35 ºC for *P. canis* CCUG 57081 detection. Then, slides were washed in a buffer solution (0.9 M NaCl, 20 mM Tris–HCl, pH 7.2, 0.1% SDS) at the same temperatures during 15 min, mounted in Vectashield Mounting Medium (Vector Laboratories, USA) and vis-ualized by fluorescent microscopy at 1000X (objective HCX PLAN APD) in a Leica DMR microscope (Leica Microsystems Lda., Lisbon, Portugal), equipped with a mercury lamp of 100W, an I3 filter for excitation between 450 and 490 nm and a N2.1 filter for excitation between 515 and 560 nm.

**Table 3:** Specific fluorescent probes used in the FISH protocol

Bacteria	Probe sequence	Fluorochrome	Reference
*Neisseria* sp.	5’-CGGGTGAGTAACATATCGG-3’	Rhodamine	[41]
*E.* *faecalis*	5’-TTATCCCCCTCTGATGGG-3’	Fluorescein	[42]
*Corynebacterium* sp.	5’-CCGGAATTTCACAGACGACG-3’	Fluorescein	[43]
*Porphyromonas* sp.	5’-TGTCAGTCGCAGTATGGCAA-3’	Fluorescein	[44]
*Peptostreptococcus* sp.	5’-TGCGCAAGCATGAAA-3’	Rhodamine	[45]

### Determination of the nisin-biogel minimum biofilm inhibitory concentration (MBIC) and minimum biofilm eradication concentration (MBEC)

A 48 h polymicrobial biofilm composed by five bacterial species was established as previously described, and its susceptibility to the nisin-biogel was determined using a modified version of the Calgary Biofilm Pin Lid Device. Gluconate chlorhexidine was used as control, based on its current use in PD control [24, 37]. MBIC and MBEC determinations were performed as described by Cunha et al. (2018) [1] with some modifications. After polymicrobial biofilm formation, pegs were washed three times with 0.9% NaCl and transferred to new 96-well plates containing 160 µL of *Brucella* broth and 40 µL of the nisin-biogel or chlorhexidine concentrations for MBIC determination. For that, the new plate was incubated for 24 h at 37 °C in microaerophilic conditions, after which the MBIC value was determined by direct observation as the lowest concentration of nisin that inhibit bacterial growth. Then, for MBEC determination pegs were washed again three times and transferred to a 96-well plate containing 200 µL of fresh *Brucella* broth medium. These plates were sealed and incubated in an ultrasonic bath, for 15 min at high frequency (50–60 Hz). Afterwards, the peg lid was replaced by a conventional one, and the plate was incubated at 37 °C for 48 h in microaerophilic conditions. After incubation, MBEC value was determined visually as the lowest concentration of nisin or chlorhexidine that eliminate microbial growth [46]. MBIC and MBEC values were confirmed by optical density measurement at 600 nm, using a microtiter plate reader (BMG Labtech, FLUOstar OPTIMA) [1].

A positive control (bacterial suspension) and a negative control (medium) were included. At the end of the experiment, the presence of the five bacterial strains in the positive controls was confirmed by FISH.

Experiments were conducted in triplicate, in independent days.

### Statistical analysis

Data statistical analysis was carried out using Microsoft Excel 2016®. Quantitative variables are expressed as mean values ± standard deviation.

## Data Availability

The datasets used and/or analysed during the current study are available from the corresponding author on reasonable request.

## Abbreviations

BHI: Brain Heart Infusion
CAS: Canine Artificial Saliva
FISH: Fluorescence In Situ Hybridization
ID: Identification
MBIC: Minimum Biofilm Inhibitory Concentration
MBEC: Minimum Biofilm Eradication Concentration
OD: Optical Density
PD: Periodontal Disease
